# Novel Treatment Strategies for Glioblastoma—A Summary

**DOI:** 10.3390/cancers13225868

**Published:** 2021-11-22

**Authors:** Stanley S. Stylli

**Affiliations:** 1 Department of Surgery, The University of Melbourne, The Royal Melbourne Hospital, Parkville, VIC 3050, Australia; sstylli@unimelb.edu.au or stanley.stylli@mh.org.au; 2 Department of Neurosurgery, The Royal Melbourne Hospital, Parkville, VIC 3050, Australia

Glioblastoma (GBM) is the most common primary central nervous system tumor in adults, accounting for approximately 80% of all brain-related malignancies [1]. It is a highly invasive disease and a paradigm of extensive intra- and intertumoral heterogeneity, presenting critical barriers to current therapies and invariably leading to treatment resistance as well as disease relapse. The current standard of care for GBM patients, involving maximal safe resection, radiotherapy and concomitant temozolomide (Stupp protocol) [2], has only provided a modest increase of 2.5 months in survival since its introduction in 2005. GBM patients have a poor prognosis, with a median survival of 12–15 months after diagnosis and a 5-year survival rate of less than 5%. Even though there have been limited advances in the progression of GBM therapeutics to significantly increase patient survival compared to other cancers, this has not dampened the motivation of researchers and clinicians to investigate novel treatment strategies for combating this disease. The Special Issue, ‘Novel Treatment Strategies for Glioblastoma’ [3], contains twelve articles (five original research articles and seven reviews) that explore a range of novel and strategic approaches for improving the treatment of GBM [4,5,6,7,8,9,10,11,12,13,14,15]. This editorial aims to briefly summarize the content of these articles.

The seven review articles focus on topics of great interest. O’Rawe et al. [9] highlight the dynamic relationship between the renin–angiotensin system (RAS), the GBM cancer stem cell niche and the tumor microenvironment (TME), and how it contributes to driving tumorigenesis and treatment resistance. They provide a concise overview on the effect of the RAS and its convergent signaling pathways on the TME, directly influencing various factors of cancer progression, including proliferation, invasion and survival. Importantly, they present data from observational and epidemiological cancer studies that involve the use of RAS inhibitors. Although the data remain inconclusive, RAS inhibitors appear to potentially be protective against cancer. They propose that existing commonly available medications can be repurposed as RAS-modulating drugs to therapeutically target the RAS in GBM, either as an alternative treatment or as an adjunct to the current standard of care.

Di Nunno et al. [10] review promising ongoing clinical trials for the treatment of primary and recurrent GBM, with a focus on novel trial design strategies. They discuss how these can be further developed in the future to streamline the testing of an ever-expanding cohort of innovative drugs to provide a tailored treatment approach for patients based on both molecular and clinical parameters. There are a number of biological obstacles that can hinder any therapeutic improvement in GBM treatment protocols, some of which are outlined in the review, including the (i) blood–brain and blood–tumor–brain barriers that impede the effective passage of therapeutic compounds to the tumor, (ii) the extensive heterogeneity of the tumor and (iii) the ability to develop/activate compensatory mechanisms to promote treatment resistance. The authors provide a summary of a number of trials for primary and recurrent GBM, but none to date have shown significant therapeutic improvements, even though there has been an increase in the molecular and biological understanding of the disease. Critical evaluation of the published results of clinical trial surveys has identified that there are well-known issues with GBM interventional trials that are terminated. These include a lack of accrual, funding problems, the absence of reliable surrogate endpoints and unbalanced patient distribution (higher numbers enrolled in the phase III component), which ultimately leads to an unpowered early efficacy study. Improvements for GBM interventional clinical trials design are presented, such as the use of ‘phase 0’ studies, which aim to target tumors with investigational agents based on the molecular profiling of the tumor coupled with an early assessment of these drugs to penetrate the blood–brain barrier.

As current treatment strategies have not delivered significant improvements in GBM patient survival, some emerging therapeutics have redirected their efforts towards reprogramming the patient’s immune system to generate an anti-tumor response. The review by Chokshi et al. [11] focuses on evaluating several immunotherapeutic approaches that have been trialed for the treatment of GBM, including various vaccination strategies, immune checkpoint inhibitors (ICIs) and chimeric antigen receptor (CAR) T cells. The exposure of tumor-associated antigens to antigen-presenting cells, which activate immune effector cells to achieve an anti-cancer immune response, form the framework of cancer vaccine functionality. Single- and multiple-antigen vaccines are presented in this review, but as they have displayed varying degrees of response, none are currently listed as being integrated into a standard of care. Immune checkpoints exist as part of a complex system of stimulatory and inhibitory regulators, with immune cells upregulating these immune checkpoints to maintain immune homeostasis and avoid autoimmunity; however, it has been determined that cancer cells can also express immune checkpoint proteins to suppress the anti-cancer immune response. Antibodies against these checkpoints, acting as ICIs, have shown great progress against melanoma and non-small-cell lung cancer, especially in blocking programmed cell death protein 1 (PD-1), which is being tested against GBM.

One of the hallmarks of cancer cell biology is an altered cell metabolism, with metabolic reprogramming occurring in cancer cells to facilitate an increase in cell proliferation, maintain self-renewal and develop treatment resistance. The review by Ghannad-Zadeh et al. [12] focuses on a one-carbon mediated de novo purine-synthesis-based metabolic pathway, summarizing the evidence supporting its role in GBM cell proliferation and tumorigenesis, as well as proposing how it can be utilized as a therapeutic modality. Alterations of this pathway have been identified in brain-tumor-initiating cells, and therefore may serve as a phenotypic marker of tumor recurrence, especially as they have a higher mitochondrial reserve than differentiated glioma cells, allowing them to use adaptive metabolic strategies to resist therapeutic stress, leading to treatment resistance. In addition, purine nucleotide synthesis has been shown to regulate DNA repair and therapeutic resistance in GBM, with increased rates of de novo nucleotide synthesis providing GBM cells with the enhanced ability to repair temozolomide (TMZ)-mediated DNA damage. As studies have demonstrated a correlation between treatment resistance and purine metabolism in GBM, the direct inhibition of purine synthesis in GBM has fueled interest in the therapeutic efficacy of this approach, including a current on-going phase 0/I trial of mycophenolate mofetil (inhibitor of IMPDH1 and GTP synthesis) in primary and recurrent GBM. Although the research is in its infancy, targeting metabolic vulnerabilities in GBM may offer an attractive strategy to overcome treatment resistance and recurrence.

Ho et al. [13] discuss the role of the gene pair |-SRGAP2–FAM72-| in the cell cycle and GBM, with the possibility of defining new therapeutic possibilities for GBM. Endogenous FAM72 expression has been detected in the hippocampal dentate gyrus, where the |-SRGAP2–FAM72-| master gene pair regulates neural stem cell (NSC) renewal, neurogenesis and brain plasticity. Alterations in the intergenic region of the two sub-gene units (gene transcription control unit) can lead to dysregulated gene expression, which may transform NSCs into cancer stem cells, leading to GBM. Through the use of gene expression data of GBM patient tumor biopsies deposited within cBioportal, the authors have demonstrated that a strong correlation exists between high FAM72 expression and the highly mutated gene signatures (EGFR, TP53, NF1, SPTA1, PIK3CA or SCN9A, MXRA5, ADAM29, KDR, PIK3C2G and LRP1B), which can lead to cell cycle activation, cell transformation and proliferation. They propose that FAM72 is an attractive target for therapy, as it is a proliferative marker expressed in the late G2M phase of the cell cycle and exhibits low expression in normal, non-neuronal tissue.

Bozzato et al. [14] highlight how nanomedicines are being investigated as an alternative treatment approach for GBM in an attempt to overcome the limitations of conventional chemotherapies, such as the lack of tumor cell specificity, toxic side effects and low biological stability. Nanomedicines can bypass some of these limitations through the encapsulation of drugs in nanosized carriers (protecting them from degradation and reducing off-target side effects in the patient) and the ability to modify the surface of the nanocarrier with targeting moieties, allowing for easy transport through the BBB or the recognition of GBM or glioma stem cells. Importantly, the use of nanocarriers as a drug delivery system can also reduce the efflux of free drugs, as the nanomedicines enter the tumor cells through the process of endocytosis via endo-lysosomal trafficking, whereas free drugs enter through diffusion, which can be located near efflux pumps.

The manuscript by Mozhei et al. [15] reviews the idea of using viral vectors either as cytotoxic agents or gene delivery tools for the treatment of GBM and provides a concise summary of molecular strategies and current clinical trials, concluding that approaches based upon targeting a specific biochemical pathway or mutation will ultimately lead to failure due to the high genomic instability and clonal selection characteristics of GBM. However, they do suggest that engaging the immune system to induce an anti-tumor response should be explored further, or alternatively that a system should be designed which irreversibly targets dividing tumor cells and not quiescent brain cells. They present an extensive list of viral vector types that have been used in gene-therapy-based clinical trials for GBM and an overview of studies that have investigated vectors based on viral backgrounds, such as adenovirus, herpes simplex, reovirus, parvovirus and poliovirus. Importantly, they indicate that a major factor which will determine the success of viral gene therapy is the physical access of the virus to the GBM cells. 

The five original articles in this Special Issue outline the innovative approaches and methodologies that research groups are utilizing to uncover novel treatment strategies for the treatment of GBM. Shapovalov et al. [8] used a genome-wide drug-induced gene expression (DIGEX) approach to define the cellular drug response phenotypes of two drugs, Mardepodect and Regorafenib, with three human GBM cell lines—U87MG, A172 and T98G. Employing a DIGEX approach allowed them to reposition the schizophrenia drug, Mardepodect, as a possible antiproliferative candidate for GBM, against Regorafenib, a drug which is already in clinical trials for GBM. The study was performed with a Clariom S Human Array, yielding more than 20,000 genes, which were linked to 18,316 identifiable protein-coding genes after being mapped to their Entrez IDs. They employed a dedicated analysis pipeline using UniProt, Entrez, Gene Ontology, the Pharos database, Reactome pathway and gene network analysis, focusing on the 200 genes with the most elevated or lowered gene expression levels and their corresponding subsets. They observed that both drugs upregulated genes encoding for specific growth factors, transcription factors, cellular signaling molecules and cell surface proteins, in addition to downregulating a broad range of targetable cell-cycle- and apoptosis-associated genes. The significant outcome of this approach is that it allowed for the detection of upregulated genes encoding for therapeutic targets of existing FDA-approved drugs, but also uncovered targets for which there are no approved drugs that may be future novel druggable targets as part of a chemistry-led discovery campaign. This approach provides a comprehensive phenotypic landscape for visualizing complex drug responses following the treatment of GBM cells, and shows that diagnosing and targeting GBM cellular phenotypes before and after drug treatment should be adopted as part of a personalized therapy program, given the pharmacological plasticity displayed by such an extremely heterogeneous disease as GBM.

As angiogenesis and apoptosis play key roles in the development of GBM, the study by Scuderi et al. [7] focused on the modulation of these two processes as a possible strategy to combat GBM progression. They used an inhibitor of the prolyl-oligopeptidase KYP-2047, which is known to modulate angiogenesis, in a series of in vitro experiments with various human GBM cell lines and in vivo experiments with a subcutaneous U87 xenograft model. They demonstrated that KYP-2047 treatment of the mice resulted in a reduced tumor burden, and that immunohistochemical studies of tumor sections revealed reduced expression of vascular endothelial growth factor, angiopoietins and endothelial-nitric-oxide synthase. The in vitro studies showed that KYP-2047 treatment was able to reduce GBM cell viability, which was coupled with an increased expression of the pro-apoptotic protein, Bax, p53 and caspase-3, and a reduction in Bcl-2.

Integrin ανβ3 receptors are overexpressed in a number of different cancers, including GBM, especially at the tumor margins (invasive regions) and blood vessels within the tumor, facilitating tumor cell motility and invasion through interactions with the extracellular matrix. It is known that the extracellular domain of integrin ανβ3 contains a novel small-molecule binding site, and the authors in the study by Godugu et al. [5] synthesized a number of high-affinity thyrointegrin ανβ3 antagonists to investigate their therapeutic efficacy against primary human GBM cell lines and the commercially available U87 cell line, using both in vitro experiments and a subcutaneous xenograft model. They observed that all antagonists were able to reduce GBM cell viability, in addition to driving a decrease in angiogenesis. Importantly, treatment with the antagonists resulted in the reduced growth of subcutaneous tumors in a U87 xenograft model, as determined by tumor volume and weight.

Schmitt et al. [6] undertook a study investigating the impact of CK2 (a ubiquitously expressed, constitutively active serine/threonine kinase) on nerve/glial antigen (NG)2 in GBM, as both have been shown to be highly expressed in GBM, determined by examination of TCGA glioma datasets. Inhibition of CK2 via a CRISPR/Cas9-mediated knockout approach or the use of a pharmacological compound, CX-4945, significantly reduced NG2 gene and protein expression in GBM cells, but also resulted in a decrease in cell proliferation and migration. Notably, they also demonstrated that CX-4945 reduced NG2 expression in patient-derived GBM cells, indicating that CX-4945 should be investigated further in preclinical studies.

Finally, as there has been evidence linking ion channels in cancer cells to a pro-invasive phenotype, and also that invadopodia as cancer-cell-based structures function to degrade the ECM and facilitate the invasive capacity of the cells, Dinevska et al. [4] performed a screening of FDA-approved ion channel drugs (that have not been previously used for the treatment of GBM patients) for their ability to have a dual impact on GBM cells; firstly, by reducing cell viability (cytotoxic effect), and secondly, by diminishing their invasive capacity (by eliciting an anti-invadopodia effect). The initial screening examining the impact of FDA-approved ion channel drugs on cell viability resulted in three drugs, flunarizine dihydrochloride, econazole nitrate and quinine hydrochloride dihydrate, being explored further for their impact on invadopodia activity. Treatment of the GBM cell lines with the three drugs demonstrated a reduction in MMP-2 secretion and invadopodia activity in comparison to the untreated GBM cells. However, the most significant observation was the reduction in radiation/temozolomide-induced invadopodia activity in the GBM cells, as radiation and temozolomide treatment forms part of the standard of care for GBM patients [2], indicating that these drugs could potentially be incorporated into current treatment to target the enhanced invasive ability of GBM cells that survive this treatment.

In summary, this Special Issue contains a set of multidisciplinary contributions that utilize various techniques and methodologies to investigate novel treatment strategies for GBM. As Guest Editor, I wish to thank all of the authors for their involvement in the Special Issue, but more importantly for tackling this challenging disease with the intention of potentially providing alternative therapeutic strategies for GBM patients in the future, which could significantly improve patient outcome.
